# Toxicomethylomics revisited: A state-of-the-science review about DNA methylation modifications in blood cells from workers exposed to toxic agents

**DOI:** 10.3389/fpubh.2023.1073658

**Published:** 2023-02-20

**Authors:** Octavio Jiménez-Garza, Manosij Ghosh, Timothy M. Barrow, Lode Godderis

**Affiliations:** ^1^Health Sciences Institute, Autonomous University of Hidalgo State, Pachuca Hidalgo, Mexico; ^2^Environment and Health Department, Katholieke Universiteit Leuven, Leuven, Belgium; ^3^Faculty of Health Sciences and Wellbeing, University of Sunderland, Sunderland, United Kingdom

**Keywords:** epigenetics, occupational toxicology, DNA methylation, biomarker of effect, mitochondrial DNA methylation, DNA repair genes, peripheral blood

## Abstract

**Introduction:**

Epigenetic marks have been proposed as early changes, at the subcellular level, in disease development. To find more specific biomarkers of effect in occupational exposures to toxicants, DNA methylation studies in peripheral blood cells have been performed. The goal of this review is to summarize and contrast findings about DNA methylation in blood cells from workers exposed to toxicants.

**Methods:**

A literature search was performed using PubMed and Web of Science. After first screening, we discarded all studies performed *in vitro* and in experimental animals, as well as those performed in other cell types other than peripheral blood cells. Results: 116 original research papers met the established criteria, published from 2007 to 2022. The most frequent investigated exposures/labor group were for benzene (18.9%) polycyclic aromatic hydrocarbons (15.5%), particulate matter (10.3%), lead (8.6%), pesticides (7.7%), radiation (4.3%), volatile organic compound mixtures (4.3%), welding fumes (3.4%) chromium (2.5%), toluene (2.5%), firefighters (2.5%), coal (1.7%), hairdressers (1.7%), nanoparticles (1.7%), vinyl chloride (1.7%), and others. Few longitudinal studies have been performed, as well as few of them have explored mitochondrial DNA methylation. Methylation platforms have evolved from analysis in repetitive elements (global methylation), gene-specific promoter methylation, to epigenome-wide studies. The most reported observations were global hypomethylation as well as promoter hypermethylation in exposed groups compared to controls, while methylation at DNA repair/oncogenes genes were the most studied; studies from genome-wide studies detect differentially methylated regions, which could be either hypo or hypermethylated.

**Discussion:**

Some evidence from longitudinal studies suggest that modifications observed in cross-sectional designs may be transitory; then, we cannot say that DNA methylation changes are predictive of disease development due to those exposures.

**Conclusion:**

Due to the heterogeneity in the genes studied, and scarcity of longitudinal studies, we are far away from considering DNA methylation changes as biomarkers of effect in occupational exposures, and nor can we establish a clear functional or pathological correlate for those epigenetic modifications associated with the studied exposures.

## Introduction

In 2011, Szyf (1), in a very illustrative and synthetic work, coined the term “toxicomethylomics.” He discussed the correlation of DNA methylation and gene expression in different genomic regions, including so-called “regulatory sequences” as well as intragenic or gene body sequences, informing future studies of human populations exposed to chemicals with mutagenic or genotoxic properties. At the time of its publication, around five research papers had been published exploring DNA methylation changes in occupationally exposed populations. In the subsequent decade, there were significant advancements in the technologies used to study DNA methylation, progressing work in the field from those analyzing methylation of specific genes to epigenome-wide analysis using microarray technology and high-throughput sequencing platforms that interrogate DNA methylation at single-nucleotide resolution. Here, we narratively review publications on DNA methylation in peripheral blood cells from people occupationally exposed to toxic agents to evaluate the insight that has been gained and the direction of the field.

## Methods

**Figure 1:**
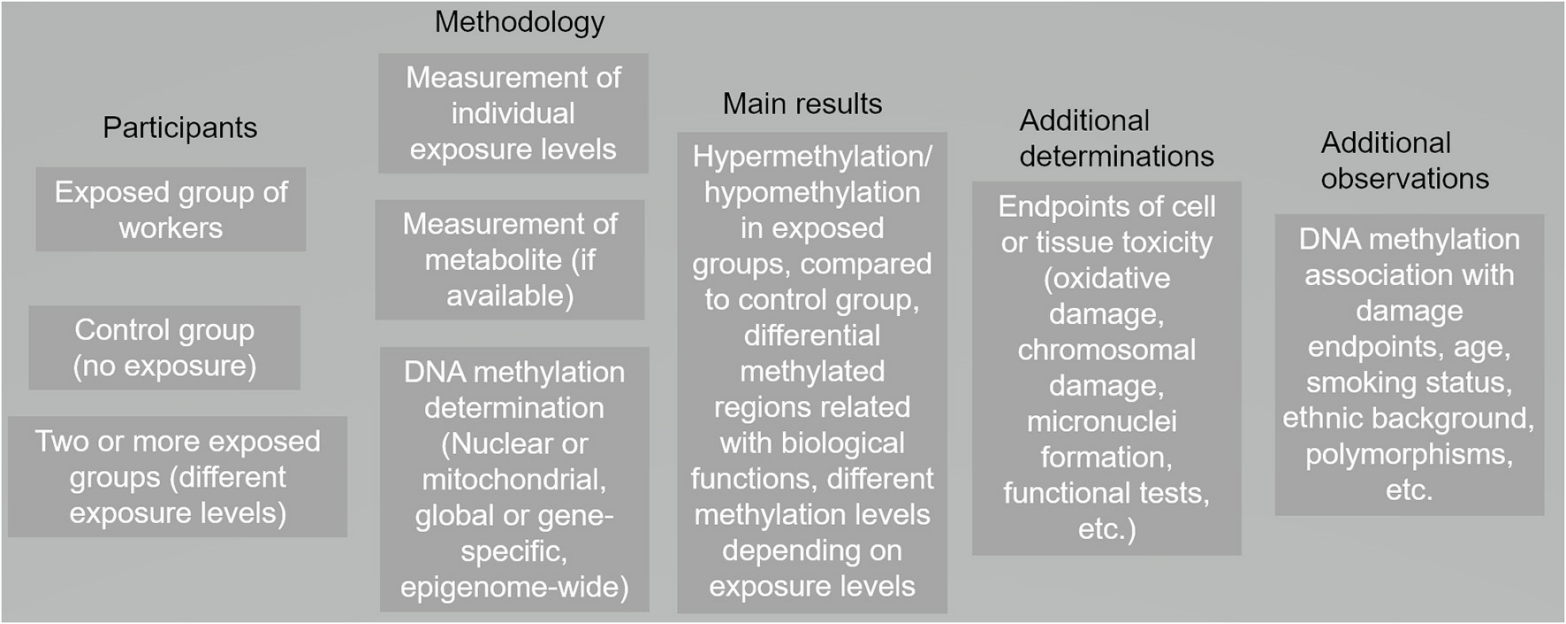
Panorama of the general design and results in toxicomethylomics studies in blood cells from occupationally–exposed individuals published from 2007 to 2022 (116 research papers).

A literature search was performed using PubMed and Web of Science. We established two searches: one with the keywords “DNA methylation” and “occupational exposure” and another search with key words “DNA methylation” and “workers.” The searches were performed between August 2022 and October 2022. After first screening (yielding 150 research papers), we read every abstract to determine if the work was performed in blood cells from healthy workers, discarding all *in vitro* and experimental animal studies as well as those performed in other human cell types; we also discarded duplicated papers. From the selected abstracts, we read the whole paper, identifying the DNA methylation technique utilized in the manuscript, the genomic target(s) (including mitochondrial DNA) and the main findings. Studies matching our inclusion criteria were entered into a database and summarized. Typically, DNA methylation research is performed describing the exposed participants, the disease/consequences of that exposure, the analysis platform for studying DNA methylation, and other endpoints such as impact upon gene expression (Figure 1). We categorized the included publications according to the compound studied. For those studies where a diverse range of compounds were analyzed, the papers were categorized according to the task/job (i.e., hairdressers, firefighters).

## Results

One hundred and sixteen research papers met the aforementioned criteria, which were each published between 2007 and 2022. These studies addressed a total of 30 different compounds and occupations. A summary of the studies characteristics is shown in Table 1. In the following section, we review their main findings according to the exposure type, describing the results according to the publication date (from the oldest to the most current). Appearance of results try to follow an order according to the chemical similarity of the compounds (organic compounds, material obtained from combustion, metals etc.). Before the section “miscellaneous” which groups studies where only one research report has been performed for each toxicant, we grouped studies performed in occupationally-exposed groups; such studies did not mention a specific exposure, but, since they were performed in peripheral blood in a group of workers, they accomplished our inclusion criteria (Summary is presented in Table 2).

**Table 1:** General characteristics of the research papers selected in the review.

**Chemical exposure/occupational group**	**Methylation platform**	**Type of DNA/region studied**	**Most frequent genes/repeated elements studied**	**Most frequent results**
Benzene/22 papers (18.9%)	Bisulfite PCR pyrosequencing 50 papers (43.3%)	Nuclear DNA 111 papers (95.6%)	LINE-1 (18 papers)	Global hypomethylation (38 times)
PAH's/18 papers (15.5%)	Methylation specific-PCR 16 papers (15.09%)	Mitochondrial DNA 5 papers (4.3%)	*p16* (15 papers)	Gene-specific hypermethylation (36 times)
PM's/12 papers (10.3%)	HPLC 7 papers (6.6%)		*MGMT* (11 papers)	
Lead/10 papers (8.6%)	850K methylation array 7 papers (3.7%)		*GSTP1* (11)	
Pesticides/9 papers (7.7%)	450K methylation array 7 papers (5.6%)		*p53* (7 papers).	
Radiation/5 papers (4.3%)	ELISA 4 papers (3.7%)			
VOC's/5 papers mixtures (4.3%)	EpiTYPER (Maldi-Tof MS) 4 papers (3.7%)			
Welding fumes/4 papers (3.4%)	Methylight technique 2 papers (1.8%)			
Chromium/3 papers (2.5%)	Others 19 papers (9.4%)			
Toluene/3 papers (2.5%)				
Firefighters/3 papers (2.5%)				
Coal/2 papers (1.7%)				
Hairdressers/2 papers (1.7%)				
Nanoparticles/2 papers (1.7%)				
Vinyl chloride/2 papers (1.7%)				
Others (one article per compound)/17 papers (14.6%)				

One hundred sixteen papers reached selection criteria.

**Table 2:** Summary of mean findings according to DNA methylation changes^*^.

**Compound/occupational group**	**Main findings (DNA methylation changes)**	**References**
Benzene	Hypomethylation in *Alu* and *LINE-1, MAGE-1* hypomethylation, *p15* (promoter region) hypermethylation; *p16* (promoter region) hypermethylation; *p16* hypermethlyation negatively correlated with *p16* expression; S-phenyl-mercapturic acid (SPMA) urinary levels negatively correlated with *p15* promoter methylation; mitochondrial DNA copy number associated with both LINE-1 hypomethylation and *p15* hypermethylation; *ERCC3* promoter hypermethylation, *PRKG1K, PARD3* and *EPHA8* hypermethylation, *STAT3* and *IFNGR1* hypomethylation; *MGMT* methylation negatively associated with SPMA urinary levels, *MGMT* hypomethylation; *DNMT3A* and *DNMT3B* mutations associated with global DNA methylation; *ERCC3* methylation correlated negatively with neutrophil abundance; participants with *MTHFR* polymorphisms showed lower methylation levels (global); p15INK4b hypermethylation; L1-Pa5 hypomethylation; LINE-1 methylation higher compared with levels 4 years before (longitudinal study) while *hMLH1* promoter methylation was lower; participants with polymorphisms in the base excision repair (BER) showed low global methylation; Lower mitochondrial DNA methylation, *MT-COI* was correlated with white blood cell count and also with platelet levels; 442 sites were hypermethylated, and 237 sites were hypomethylated; DNA copy number negatively correlated with global DNA methylation; *UCA1* methylation negatively correlated with benzene exposure levels; accelerated epigenetic aging in exposed workers; LINC00173 promoter methylation was negatively correlated with its expression; significant alterations in methylation at 340 CpG sites, impacting AMPk signaling pathway	(2–23)
Volatile organic compound mixtures	Global hypermethylation, methylation levels negatively associated with exposure; *IL6* hypermethylation, CYP2E1 methylation was negatively correlated with benzene exposure levels, CYP2E1 SPMA urinary levels were positive correlated; *TOP2A, SOD1, TNF-α* hypermethylation, ethylbenzene levels significantly correlated with TOP2A methylation; IL-6 hypomethylation, *TNF-α* hypermethylation (5′UTR region); highest amount of differentially methylated regions in workers exposed to a tertiary mixture	(24–28)
Toluene	CYP2E1 promoter hypermethylation in smokers (exposed to toluene); 26 genes upregulated and hypomethylated, 32 genes downregulated and hypermethylated; hypomethylation in the 5′UTR region of the CYP2E1	(27, 29, 30)
Polycyclic aromatic hydrocarbons (PAH's)	Alu and LINE-1 hypermethylation, IL-6 methylation positively correlated with PAH levels; *TP53* promoter hypermethylation; *p16(INK4α)* hypermethylation, hypermethylated CpG sites positively correlated with urinary 1 hydroxypyrene (1-OHP); DUSP22 promoter hypomethylation correlating with duration of firefighting service; global hypomethylation as well as *MGMT* hypomethylation; *TP53* hypomethylation; negative correlations between promoter DNA methylation (*IL-12* and *TP53*) with urinary 1-OHP; *p14(ARK), p15(INK4b)*, and *p16(INK4a)* hypermethylation, correlation with years worked and also with urinary 1-OHP levels; *RASSF1A, MGMT*, and *LINE-1* hypomethylation, negative correlation with markers of cytogenetic damage; *TRIM36* promoter hypermethylation, positive correlation with urinary 1-OHP; *FHL2* hypermethylation; high urinary 1-OHP corresponded to hypomethylation of LINE-1; *F2RL3* and *AHRR* hypomethylation; *AHRR* hypomethylation; *CYP1A1* hypomethylation; significant relationship between CYP1A1 hypomethylation and high 8-hydroxydeoxyguanosine (8-OHdG); *OGG1* methylation levels positively correlated with urinary 1-OHP levels; *IFN-γ* hypermethylation, *IL-10* hypomethylation, *IL-10* methylation inversely correlated with urinary 1-OHP; *AHRR* and *F2RL3* hypomethylation	(31–48)
Particulate matter (PM)	No significant differences between pre-shift and post-shift methylation levels; *APC* and *p16* hypermethylation in post-shift, *APC* methylation positively associated with PM_10_ exposure, *TP53* and *RASSF1A* hypomethylation in post-exposure; *LINE-1* elements showed a differential effect by evolutionary age; *MT-RNR1* methylation positively correlated with mtDNA copy number; inverse correlation between *NOS3* methylation exposure to PM10; *iNOS* promoter methylation was associated with increased PM 2.5 exposure; positive correlation between PM2.5 exposure and *LINE-1* methylation; *NBL2* methylation positively associated with concentrations of silicon and calcium; ambient PM_10_ negatively correlated with *NBL2* methylation; EBV-Wp hypomethylation; 13,643 differentially methylated CpG loci, groups of differentially methylated loci annotated to the same gene were linked to diabetes mellitus, respiratory diseases, the dopaminergic system of the brain and neurodegenerative diseases; negative association between methylation of platelet mitochondrial gene MT-ATP6 and PM exposure	(49–60)
Pesticides	Hypermethylation in three CpG sites; *GSTP1* hypermethylation, *MGMT* hypomethylation; global hypermethylation; global hypomethylation; global hypomethylation, *p16* promoter hypermethylation, DNA methylation levels associated with elevated oxidative stress; significant impacts upon gene expression identified for seven CpGs; *CDKN2B* hypomethylation, *CDKN2A* hypermethylation; *CDKN2B* hypermethylation	(61–69)
Lead	*p16* hypermethylation; *ALAD* hypermethylation, ALAD decreased expression; global hypomethylation, significant associated between methylation levels and blood lead levels; significant correlation between DNA methylation and lead concentrations; *hMLH1, p14, p15*, and *p16* hypermethylation; 180 differentially methylated genes; high blood lead levels associated with reduced *GCLC* and *GSTP1* methylation; differentially methylated positions (DMP) were significantly enriched in genes related to nerve conduction and cell cycle	(15, 70–76)
Chromium VI	Global hypomethylation; *MT-TF* and *MT-RNR1* hypomethylation; *OGG1, MGMT*, and *RAD51* hypermethylation, positive correlation between exposure level and methylation, negative correlation between methylation and expression	(77–79)
Welding fumes	*APC* hypermethylation; *F2RL3* hypomethylation; *MT-TF* hypomethylation; *B3GNTL1* decreased methylation overtime (longitudinal study)	(80–83)
Coal	Global hypermethylation, variants within the CYP1A1 gene associated with lower global methylation	(84, 85)
Radiation	Global hypomethylation; *GSTP1* and *p16* hypermethylation correlated with exposure; *TP53* and *SOD3* hypermethylation; global hypomethylation; global hypomethylation	(86–90)
Nanoparticles	Hypomethylation at 341 CpG sites and hypermethylation of 364; more pronounced methylation changes in exposed group over the course of four years	(91, 92)
Trichloroethylene	*TRIM68* hypomethylation; increased epigenetic age acceleration	(20, 93)
Firefighters	*YIPF6, MPST* and *PCED1B* with significant changes in methylation; *SULT1C2* hypomethylation in Hispanics; 680 CpG sites with altered methylation	(94–96)
Hairdressers	“Hair waving” was associated with less frequent *CDKN2A* methylation; carriers of genetic variants within the *FLG* gene showed higher methylation of *CDKN2A*	(97, 98)
Vinyl chloride	*MGMT* hypomethylation; 9,534 DMR's: 4,816 were hypomethylated and 4,718 were hypermethylated	(99, 100)

* We include in this summary those exposure/occupational group with more than two research papers included in our analysis.

### Benzene

The first paper regarding epigenetic effects in workers exposed to a carcinogenic compound was in people exposed to this very well-known leukemogen. In 2007, Bollati et al. (2) used pyrosequencing and reported hypomethylation of the repetitive elements *Alu* (taking its name from the *Arthrobacter luteus* restriction endonuclease) and the long interspersed nuclear element-1 (LINE-1), as a surrogate for global DNA methylation. Their work also examined methylation at the promoter region of two genes, revealing loss of methylation for the Melanoma-associated antigen 1 gene (*MAGE-1)* and hypermethylation of *p15*, a tumor repressor gene. This first report of global hypomethylation (LINE-1) in response to exposure to a carcinogenic compound has since been confirmed by a number of other studies, as has the observation of *p15* hypermethylation. Moreover, participants in this study were considered to be exposed to “low-levels,” which suggests DNA methylation as an early biological response marker even to low exposures. Five years later, Xing et al. (3) measured promoter methylation of the *p16* (also a tumor repressor gene) promoter region in benzene exposed workers. In addition, they measured expression of *p16* at the mRNA level. They reported promoter hypermethylation in the exposed group, and methylation levels negatively correlated with p16 expression; the first evidence that promoter methylation in response to benzene exposure can silence gene expression. Although examined in only a few individuals (eleven exposed vs. eight controls), those results were supported by similar observations in cell lines exposed to benzene metabolites. In the same year, Seow et al. (4) showed an interesting negative correlation between urinary S-phenyl-mercapturic acid (SPMA, a main metabolite of benzene) with methylation levels of *Alu* elements and the *p15* gene promoter, which represents a link between xenobiotic biotransformation enzyme activity and DNA methylation. Also in 2012, Carugno et al. (5) using data from the same population studied by Bollati et al. (2), described an association between mitochondrial DNA copy number and both LINE-1 hypomethylation and *p15* hypermethylation, providing evidence for the relationship between nuclear epigenetic marks and the function of mitochondria; organelles which are highly affected by toxic chemicals *via* redox imbalance.

Further, gene polymorphisms are known to influence the body's response to toxic chemicals. Hence, Xing et al. (6) investigated the relationship between single nucleotide polymorphisms (SNPs) within four genes encoding metabolic enzymes and promoter methylation of eleven genes associated with hematotoxicity in benzene-exposed workers. They observed that ERCC excision repair 3 gene (*ERCC3*) promoter methylation was increased in the exposed group, and also that individuals carrying a genetic variant mapping to Epoxide Hydrolase 1 gene (*EPHX1)* had decreased *ERCC3* methylation. In 2014, Yang et al. (7) employed the widely-used Illumina Infinium 450K methylation microarray platform for epigenome-wide analysis, which revealed three hypermethylated genes: Protein kinase, cGMP-dependent, Regulatory, Type I (*PRKG1K)*, Par-3 Family Cell Polarity Regulator (*PARD3)*, and Ephrin type-A receptor 8 (*EPHA8*) and two hypomethylated: Signal Transducer And Activator Of Transcription 3 (*STAT3)* as well as Interferon Gamma Receptor 1 (*IFNGR1*) in four benzene-exposed individuals compared to controls. Importantly, hypermethylation was shown to decrease gene expression, while hypomethylated genes showed higher expression. They concluded that aberrant hypomethylation of *STAT3* might be a potential biomarker of chronic benzene poisoning.

Other authors have tried to relate DNA damage markers with methylation, such as Li et al. (8), who described that DNA damage, as well as SPMA urinary levels, were negatively associated with O-6-Methylguanine-DNA Methyltransferase gene (*MGMT)* methylation. *MGMT* methylation was also significantly decreased in the benzene-exposed group. In the same year, Zhang et al. in an effort to elucidate the underlying mechanisms of global hypomethylation caused by benzene exposure, investigated the relationship between global methylation and several endpoints related with DNA damage (including mutations) and polymorphisms in DNA Methyltransferase 3 Alpha gene (*DNMT3A)* and DNA Methyltransferase 3 Beta *(DNMT3B)*. They described the association between SNPS within both the *DNMT3A* and *DNMT3B* genes and global DNA methylation. In that same year, Zheng et al. (9), analyzed *ERCC3* promoter methylation in benzene-exposed workers and its possible relationship with hematotoxicity markers. Methylation at 2 CpGs were correlated negatively with neutrophil abundance. Ren et al. (10), analyzed global DNA methylation and its relationship with polymorphisms within the methylenetetrahydrofolate reductase (*MTHFR*) gene, which encodes a key enzyme required for the production of methyl donors for DNMTs. Individuals homozygous for the TT allele at rs1801133 displayed lower methylation levels compared with those homozygous for the wild type allele. These findings illustrate the interaction between genetic variants and the epigenome, including variants within genes encoding the epigenetic machinery of the cell and also enzymes that may mediate response to environmental exposures. In the same year, Jamebozorgi et al. (11), described higher promoter methylation for *p15INK4b*, a cyclin-dependent kinase inhibitor gene, in workers exposed to benzene compared to controls. Looking for possible methylation changes in repetitive elements subfamilies, Rota et al. (12) reported that only L1-Pa5 was hypomethylated in petrol station workers (89 workers) compared to controls, with *Alu* subfamilies showing no such effect, in contrast to previous findings. Costa-Amaral et al. (13), did not find differences in gene-specific methylation for the Cyclin Dependent Kinase Inhibitor 2B (*CDKN2B)* and Kruppel Like Factor 6 (*KLF6)* genes in Brazilian workers exposed to benzene. In a very interesting study using a longitudinal approach, Ren et al. (14) followed benzene-exposed workers from 2009 to 2013, analyzing DNA methylation once in 2009 and again in 2013, measuring methylation of LINE-1, *MGMT*, and Human MutL homolog 1 gene (*hMLH1)*. LINE-1 methylation was higher in 2013 compared to 2009, while promoter methylation in *hMLH1* was lower and corresponded with the reduction in benzene exposure. Zhang et al. (15), studying the base excision repair (BER) pathway and their polymorphisms, in benzene-exposed workers, found that the rs1130409 variant allele (GG+GT) was associated with low global DNA methylation. Wang et al. (16) studied mitochondrial DNA methylation, specifically at the Mitochondrially Encoded Cytochrome c Oxidase I (*MT-COI*) gene, and reported two interesting results: mitochondrial methylation was lower in the exposed group; and methylation of *MT-COI* was correlated with white blood cell count and also with platelet levels. Epigenome-wide analysis was performed on a large scale for the first time by Ren et al. (17), using the Infinium 450K methylation microarray. They reported 442 sites in benzene-exposed participants were hypermethylated, corresponding to 253 genes, and 237 sites were hypomethylated, corresponding to 130 genes. Correlation analyses showed that promoter methylation of Colony Stimulating Factor 3 Receptor (*CSF3R)* and Coagulation Factor II Thrombin Receptor (*F2R)* genes were both highly correlated with gene expression. The *CSF3R* gene is of particular interest as it encodes a protein involved in the differentiation of granulocytes. In persons exposed to the highest benzene levels, in the poisoning cases, the proportion of neutrophils in the blood was significantly different by *CSF3R* methylation levels, demonstrating a potential function role for this gene in the toxicity associated with benzene exposure. By their side, Ji et al. (18) reported lower global DNA methylation in benzene-exposed workers compared to controls, with mitochondrial DNA copy number negatively correlated with global DNA methylation; and also Pan et al. (19), using methylation-specific PCR to analyze the Urothelial Cancer Associated 1 gene (*UCA1)*, found that methylation at the promoter region of this gene is negatively correlated with benzene exposure time. Together, there is a wealth of data to demonstrate both global hypomethylation and gene-specific changes in response to benzene exposure, with *CSF3R* a particularly interesting candidate. In 2022 we have data from three research works: van der Laan et al. (20) using the 450K microarray, found that occupational exposure to benzene accelerated epigenetic aging in the blood and was also associated with shorter estimated telomere length. Zhang et al. (21), studying promoter methylation of the Long intergenic non-protein coding RNA 173 (LINC00173) vs. the expression of this RNA in benzene exposed workers, found that LINC00173 promoter methylation was negatively correlated with its expression. Very recently, Phillips et al. (22), in a cross-sectional epigenome-wide association study, using the Human Methylation 450 Bead Chips, investigated differences in blood cell DNA methylation among 50 benzene-exposed subjects and 48 controls, and analyzed CpG-level and regional-level. They report genome-wide significant alterations in methylation at 340 CpG sites, and also in mean methylation levels at a large genomic region; also, Pathway analysis of genes sites revealed an impact on the AMPK signaling pathway.

### Volatile organic compounds mixtures

VOCs are chemical compounds present in a myriad of labor settings. The most widely-studied of these is benzene, which has been discussed in the previous section. However, other VOCs have been described as contributing to asthma (toluene), have been classified as carcinogenic in animals (ethylbenzene) or are reprotoxic and toxic to the nervous system (such as n-hexane). These mixtures are frequently found in organic solvents. Epigenetic effects in workers exposed to these kind of mixtures were first evaluated in 2012 by Godderis et al. (24), who evaluated DNA global methylation in workers exposed to solvents. They observed global hypermethylation in response to exposure, which was negatively associated with exposure levels. Furthermore, SNPs within the Glutathione S-Transferase Pi gene (*GSTP1)*, encoding an enzyme involved in detoxification processes, were found to be significantly associated with this global DNA hypomethylation. In 2017, Jiménez-Garza et al. (25), studying groups of Mexican workers exposed to solvents *via* work at a shoe factory or gas station in comparison to office-based controls, evaluated promoter methylation of the Interleukin-6 (*IL6)*, Cytochrome P450 2E1 (*CYP2E1)*, and Inducible Nitric oxide synthase (i*NOS)* genes. *IL6* promoter methylation was higher in gas station attendants (the group with the highest exposure levels to benzene), while *CYP2E1* promoter methylation was negatively correlated with benzene exposure levels. Furthermore, positive correlations were observed between *CYP2E1* promoter methylation and both *iNOS* promoter methylation and urinary levels of SPMA. A year later, the same group of researchers (26) studied promoter methylation for a panel of genes including Cyclooxygenase II (*COX-2), GSTP1*, Heme Oxygenase 1 (*HMOX-1), iNOS*, Superoxide Dismutase 1 (*SOD1)*, Tumor necrosis alpha (*TNF-*α*)*, and Topoisomerase II alpha (*TOP2A)*. This time they added another group for comparison: shoe workers exposed to very high solvent levels, excepting benzene. Hypermethylation of *TOP2A, SOD1, TNF-*α was observed among these shoe workers. Further to their previous observations of correlations between epigenetic changes of different genes, significant correlations were observed between *GSTP1* promoter methylation and methylation of both *iNOS* and *COX-2*. Ethylbenzene exposure levels were significantly correlated with *TOP2A* methylation. Interestingly, a “cumulated time of exposure” showed significant negative correlation with methylation of both *TNF-*α and *iNOS*. In a subset of workers taken from the two latter mentioned studies, Jiménez-Garza et al. (27) and now studying methylation mapping to the 5′UTR region of genes, analyzed methylation of the *CYP2E1, IL-6, SOD1*, and *TNF-*α genes. They reported lower methylation levels for *IL-6* and higher methylation levels for *TNF-*α for the mixtures-exposed group compared to controls. Recently, Yu et al. (28), studied genome-wide DNA methylation in blood from Korean workers exposed to a binary mixture (toluene and xylene) and compared results with workers exposed to a tertiary mixture (toluene, xylene, and ethylbenzene group). Authors observed the highest amount of differentially methylated regions in the group exposed to the tertiary mixture, which underlines the complexity of studying the effects of a mixture of different compounds and making appropriate inferences.

### Toluene

Toluene is a VOC that is frequently used in many industries. Toluene chronic exposure has been related with respiratory affections and, when combined with other VOCs, may increase their hematotoxic effects. To date, only three studies have examined DNA methylation in response to exposure to this compound. In 2015, Jiménez-Garza et al. (29) analyzed promoter methylation of *COX-2, CYP2E1, GSTP1, HMOX-1, IL-6, iNOS, SOD1, TNF-*α, and *TOP2A* in Mexican workers from a tannery who are exposed to toluene. While they did not report statistically significant differences between exposed individuals and controls, positive correlations were observed between toluene airborne levels and both *CYP2E1* and *IL6* promoter methylation. *CYP2E1* promoter methylation levels were higher in toluene-exposed smokers compared to non-smokers, and were also correlated with *GSTP1* and *SOD1* promoter methylation. One year later, Hong et al. (30) studied DNA methylation in Korean workers exposed to toluene. They reported 26 genes that were upregulated and hypomethylated, as well as 32 genes downregulated and hypermethylated in the exposed group. In 2020, studying a subset of the toluene-exposed workers from their 2015 publication, Jiménez-Garza et al. examined 5'UTR methylation of five genes: *CYP2E1, IL-6, SOD1*, and *TNF-*α. They observed hypomethylation in the 5′UTR region of the *CYP2E1* gene in toluene-exposed workers compared to controls, further underlining the apparent importance of this gene in our understanding of the effects of toluene exposure.

### Polycyclic aromatic hydrocarbons

There are different labor activities where workers are exposed to PAHs. Many of these compounds have carcinogenic properties, with benzo[a]pyrene specifically causing lung cancer, while other PAHs such as naphthalene, acenaphthylene, acenaphthene, fluorene, anthracene, phenanthrene, fluoranthene, pyrene, chrysene, benz[a] anthracene, benzo[b]fluoranthene, benzo[k] fluoranthene, B[a]P, indeno[1,2,3-cd]pyrene, benzo[g,h,i]-perylene, and dibenz[a,h]anthracene have also shown carcinogenic potential. The first study we identified was that of Pavanello et al. (31), who investigated coke-oven workers exposed to PAHs. They studied methylation of the *p53, p16*, Hypermethylated in cancer 1 protein (*HIC1)*, and *IL-6* genes as well as of *Alu* and LINE-1 repetitive elements. They observed *Alu* and LINE-1 hypermethylation in the exposed workers, while gene-specific *IL-6* methylation was significantly and positively correlated with PAH exposure levels. The next year, the same research group (32), studied the same population of coke-oven workers and reported *TP53* promoter hypermethylation in response to exposure, with *TP53* hypomethylation also demonstrated to be one of the principal determinants of shorter telomere length. In 2012, Yang et al. (33) examined 69 workers exposed to PAHs, and specifically focused upon *p16* (*INK4*α) promoter methylation. They reported *p16* (*INK4*α) hypermethylation in exposed workers, and they also observed that hypermethylated CpG sites were positively correlated with levels of urinary 1 hydroxypyrene (1-OHP) and with the frequency of cytokinesis block micronucleus. That same year, Ouyang et al. (34), studied promoter methylation of the *GSTP1*, Interferon Gamma (*IFN-g)*, RAD21 cohesin complex component (*RAD21)*, and Dual Specificity Phosphatase 22 (*DUSP22)* genes in firefighters exposed to PAHs. Firefighters had a higher prevalence of *DUSP22* promoter hypomethylation in the blood, and the extent of hypomethylation correlated with duration of firefighting service. One year later, Duan et al. (35), examined global methylation (using LINE-1 as a surrogate marker) and promoter-specific methylation of the *MGMT* gene in 82 PAH-exposed workers. They reported global hypomethylation as well as hypomethylation of *MGMT*. In addition, LINE-1 methylation was inversely associated with DNA damage as determined by Comet assay, and a significant increase of micronuclei in the low *MGMT* methylation group. Pavanello et al. (36), studying *TP53* methylation in Polish workers exposed to PAHs, found hypomethylation of the *TP53* promoter, contrary to their previous study of 2010. That same year, Alegría-Torres et al. (37), studied promoter methylation of five genes: *TP53, TNF-a, IFN-c, IL-6*, and Interleukin 12 (*IL-12*) in Mexican brickmakers. They did not find any difference in promoter methylation of these genes, but they reported negative correlations between promoter DNA methylation of two genes (*IL-12* and *TP53*) with urinary 1-OHP concentration; a metabolite from PAH exposure.

In 2015, Zhang et al. (38) examined methylation of the *p14*(*ARK*), *p15*(*INK4b*), and *p16*(*INK4a*) genes in coke oven workers. The three genes studied were hypermethylated, with two of these correlated with years worked and also with urinary 1-OHP levels. The next year, Zhang et al. (39) examined promoter methylation of the *p16*, Ras Association Domain Family Member 1 (*RASSF1A)* and *MGMT* genes, as well as LINE-1, in workers exposed to diesel exhaust. The exposed group showed lower methylation levels for all three genes compared to controls, with methylation levels negatively correlated with markers of cytogenetic damage. In 2017, He et al. (40) found that Tripartite Motif Containing 36 gene (*TRIM36)* promoter hypermethylation was positively correlated with the level of urinary 1-OHP in 151 exposed workers. One year later, Li et al. (41) analyzed epigenome-wide methylation in Chinese participants exposed to different levels of PAHs (including an occupationally exposed group from coke-oven emissions) using the 450 k microarray. They found hypermethylated CpGs mapping to the Four And A Half LIM Domains 2 (*FHL2)* gene, as well as gene expression positively associated with methylation. In the same year, (47) investigated global and *AHRR* promoter methylation in coke oven workers. They observed that the workers with high urinary 1-OHP have hypomethylation of LINE-1. Heavy smoking was associated with a significantly increased risk of hypomethylation of Aryl-Hydrocarbon Receptor Repressor (*AHRR)* gene, in line with numerous other reports of *AHRR* serving as an effective biomarker of tobacco smoke exposure. Alhamdow et al. (42) studied F2R like thrombin or trypsin receptor 3 (*F2RL3)* and *AHRR* (exon 1 region) in chimney sweeps from Sweden. In these two genes that are known to be associated with tobacco smoke exposure, they found hypomethylation of both in the exposed group, and that non-smoking chimney sweeps had lower average *F2RL3* methylation levels. Wahlberg et al. (43) similarly studied *AHRR* and *F2RL3* in chimney sweeps and again reported *AHRR* hypomethylation, but they also found that variants within the Filaggrin (*FLG)* gene were associated with ~2.5% higher methylation of *F2RL3*. Liu et al. (44) examined methylation at the promoter region of Cytochrome P450 1A1 (*CYP1A1)* gene in Chinese coke-oven workers, with hypomethylation observed in workers highly exposed to PAHs and a significant relationship between *CYP1A1* hypomethylation and high 8-hydroxydeoxyguanosine (8-OHdG); a specific marker of oxidative stress. In a third publication from that year, Fu et al. (45), also studying Chinese coke oven workers, examined 8-oxoguanine DNA glycosylase (O*GG1)* gene promoter methylation and described its positive correlation with urinary 1-OHP levels. In the two most recent papers, both from 2020, Zhao et al. (46) reported *IFN-*γ hypermethylation and *IL-10* hypomethylation in coke-oven workers, with *IL-10* methylation inversely correlated with urine 1-OHP concentration. Finally, Alhamdow et al. investigating exposure to high-molecular-weight PAHs in chimney sweeps, confirmed hypomethylation of *AHRR* and *F2RL3* in exons 1 and 2, respectively.

### Particulate matter

Exposure to PM is associated with increased risk of asthma and cardiovascular disease. Although PM is present in high levels in the ambient air of polluted cities, some workers are continuously exposed to these particles in the occupational setting. Most of the studies investigating epigenetic changes in occupational cohorts exposed to PM come from the same research groups, evaluating different endpoints and contrasting the results obtained in Italian steel workers and Chinese truck drivers. Two studies were published in 2011 where Italian steel workers were studied. Dioni et al. (49) measured Human Telomerase Reverse Transcriptase (*hTERT)* methylation in peripheral blood, comparing pre-shift methylation levels with the post-shift changes in the same workday (with no control group), but no significant differences were observed. The other report from that year is the one from Hou et al. (53) who investigated, also in steel workers, promoter methylation levels of four tumor suppressor genes: Adenomatous polyposis coli (*APC)*; *p16*: *TP53;* and *RASSF1A*. They also compared methylation before and after shifts, with mean promoter methylation levels of *APC* and *p16* significantly higher in post-exposure samples compared to baseline samples. Furthermore, *APC* methylation was positively associated with PM_10_ exposure. Conversely, the mean levels of *TP53* and *RASSF1A* promoter methylation were significantly decreased in post-exposure samples.

Two years later, Byun et al. (59) studied methylation in 10 repetitive element subfamilies by their evolutionary origins in three different groups of workers occupationally exposed to PM: steel workers (the aforementioned Italian steel workers); gas station attendants; and Chinese truck drivers (120 workers in total). LINE-1 elements showed a differential effect by evolutionary age, with older elements showing greater hypomethylation in response to PM exposure. That same year, Byun et al. (60) examined mitochondrial DNA methylation and reported that participants with high metal-rich PM_10_ exposure showed higher Mitochondrially Encoded tRNA Phenylalanine (*MT-TF)* and Mitochondrially Encoded 12S RRNA (*MT-RNR1)* methylation in comparison to control subjects, with *MT-RNR1* methylation positively correlated with mtDNA copy number. In the same year, Tarantini et al. (50) studied promoter methylation of the Nitric Oxide Synthase 3 (*NOS3)* and Endothelin 1 (*EDN1)* genes in steel workers and reported an inverse correlation between *NOS3* methylation exposure to PM_10_, while *EDN1* methylation showed a similar negative correlation with zinc exposure. Kile et al. (51), studying boilermaker welders, examined *iNOS* promoter methylation, assessing pre- and post-shift changes. They reported that PM_2.5_ exposure was associated with increased methylation of this gene, with a positive association between levels and the number of years worked as a boilermaker. In 2014, Fan et al. (52) measured methylation of repetitive elements in the genome in boilermakers exposed to high levels of PM_2.5_. They reported no differences in pre-shift and post-shift methylation of tandem repeats but did observe a positive correlation between PM_2.5_ exposure and LINE-1 methylation. In another study of truck drivers in Beijing, Hou et al. (53) measured methylation of three tandem repeat elements in relation to exposure to different components of PM. *NBL2* methylation (a DNA repeated sequence) was positively associated with concentrations of silicon and calcium, while in office workers *SAT*α methylation (Satellite alpha, a repetitive element) was positively associated with sulfur. From a similar study performed in the same population, Guo et al. (54) reported the effects of short-term exposure to inhalable particulate matter, and again focused upon tandem repeat sequences within the genome. Both personal PM_2.5_ and ambient PM_10_ levels showed an effect upon *SAT*α methylation that was stronger among higher-exposed truck drivers than office workers, while ambient PM_10_ was negatively correlated with *NBL2* methylation. In 2017, Mercorio et al. (55) studied 63 male workers from an electric furnace steel plant and examined methylation of the Wp promoter of the Epstein-Barr Virus (EBV-Wp) and the promoter of the human-endogenous-retrovirus w elements (HERV-w). They reported EBV-Wp hypomethylation following exposure, yet PM and metal components were shown to exhibit a positive effect upon EBV-Wp methylation. We detected two important studies in 2022: first, Honkova et al. (56) studied policeman exposed to different levels of PM 2.5, benzo[a]pyrene and NO_2_ from three different cities; DNA methylation was analyzed by a genome-wide microarray method. They describe 13,643 differentially methylated CpG loci between policeman from two different cities; they also found two hypomethylated loci annotated to the DNA repair gene *XRCC5*; groups of differentially methylated loci annotated to the same gene were linked to diabetes mellitus, respiratory diseases, the dopaminergic system of the brain and neurodegenerative diseases. Finally, Guo et al. (57), investigated mitochondrial DNA (mtDNA) methylation in workers from a coke-oven plant exposed concurrently to PAH's and PM 2.5 mtDNA methylation was measured by bisulfite pyrosequencing of two genes of ATP synthase (*MT-ATP6* and *MT-ATP8*). They observed a significant, negative association between methylation of platelet mitochondrial gene *MT-ATP6* and PM exposure.

### Pesticides

There are a wide variety of chemical compounds that function as pesticides. Acute health effects are common in highly exposed people, but chronic exposures are also known to have serious detrimental effects upon human health. In 2016, the first report on farm workers exposed to pesticides associated with DNA methylation appeared: Howard et al., using the 450k microarray platform, identified three CpG sites that are hypermethylated in exposed farmworkers compared to non-farmworkers following Bonferroni correction, suggestive of a general decrease in gene expression of the regulated genes. Pathway analysis, incorporated 33 other CpG sites that showed significant changes between the exposure groups that did not survive Bonferroni correction, particularly revealed enrichment in genes implicated in immune function, as well as growth hormone signaling and DNA damage response. One year later, Rusiecki et al. (63) analyzed methylation at the promoter region of three genes: Cadherin 1 (*CDH1), GSTP1, MGMT*, and LINE-1 (as a surrogate marker of global methylation) in pesticide applicators from the USA. They categorized results according to having a high pesticide exposure event (HPEEs), with those individuals who have ever experienced an HPEE (*n* = 142), associated with hypermethylation of the *GSTP1* promoter and loss of methylation at the *MGMT* promoter, with greater effects observed in applicators of more advanced age and with lower plasma folate levels. In 2018, Benedetti et al. (64) published their findings in 137 farmers exposed to a pesticide mixture, which revealed global hypermethylation in the exposed group, as determined by high performance liquid chromatography rather than the more widely-used LINE-1 assay. In direct contrast to this, Benitez-Trinidad et al. (65) observed LINE-1 hypomethylation in exposed workers (urban sprayers; *n* = 190). The latter is supported by Kahl et al. (62), who similarly reported global hypomethylation, in addition to *p16* promoter hypermethylation, in pesticide-exposed tobacco farmers. Importantly, they reported that global DNA methylation levels were associated with elevated oxidative stress, which may help to bring mechanistic insight into the relationship between exposure, the epigenome and human health. In a similar population, Kahl et al. (61) examined global DNA methylation in relation to nutritional intake, but they did not identify significant associations. The fourth study published in 2018 came from van der Plaat et al. (66) who, using Illumina 450K microarrays, described methylation changes associated with high pesticide exposure at 31 CpGs mapping to 29 genes. Significant impacts upon gene expression were only identified for seven of these, which may suggest a broader impact upon the epigenome than locus-specific modulation of gene expression. In 2019, Herrera-Moreno et al. (67) looked for methylation changes within the 5′UTR region of the *CDKN2B* gene and the promoter of Cyclin Dependent Kinase Inhibitor 2A (*CDKN2A)* in Mexican workers exposed to pesticides. *CDKN2B* hypomethylation was observed in exposed individuals, with a signification association between methylation and exposure levels, while hypermethylation of the *CDKN2A* promoter was observed in moderately-exposed individuals. Finally, Paredes-Céspedes et al. (68), determined methylation profiles of the *CDKN2B* and *CDKN2A* genes in a genetically conserved population exposed to pesticides. They observed that the farmer group presented a higher methylation percentage of *CDKN2B* than the non-farmer group, but no differences in CDKN2A were observed between groups. A positive correlation between methylation of CpG site 3 of *CDKN2B* and time working in the field was observed in the farmer group.

### Lead

Lead is a toxic metal with adverse health effects, particularly to the nervous system. Indeed, according to the World Health Organization, lead exposure accounted for more than one-million deaths in 2017. The first report of epigenetic changes in occupationally-exposed individuals is from 2010, when Kovatsi et al. (70) analyzed *p16* promoter methylation in nine exposed workers. Although they used a qualitative approach to exposure, *p16* promoter hypermethylation was apparent among exposed workers. Li et al. (71) studied methylation of the Aminolevulinate Dehydratase (*ALAD)* gene promoter in 103 workers from a battery plant. This gene encodes an enzyme whose activity is inhibited by lead and can thereby result in anemia. The study revealed *ALAD* hypermethylation in exposed workers and a concurrent decrease in gene expression, with *ALAD* methylation associated with increased risk of lead poisoning. Together, these findings have brought mechanistic insight into the inhibition of normal ALAD activity through lead exposure. In 2013, Li et al. (72) examined LINE-1 methylation in 53 exposed battery plant workers and 57 age-matched controls and reported hypomethylation in exposed participants, with a significant associated between methylation levels and blood lead levels. One year later (101), the same group performed further analysis within the same cohort to examine *GSTP1* promoter methylation, but no statistically significant differences were present. In 2017, Devóz et al. (73) analyzed global methylation in Brazilian workers employed in automotive battery factories, which further confirmed a significant correlation between DNA methylation and blood/plasma lead concentrations. A year later, Yu et al. (74) analyzed the methylation of six human tumor suppressor genes, four of which were hypermethylated (*hMLH1, p14, p15*, and *p16*) in the exposed group, while two (*GSTP1* and *MGMT*) showed no significant changes. In 2019, epigenome-wide analysis through the expanded Illumina Infinium EPIC (850K) microarray platform was performed by Zhang et al. (15) who identified 180 differentially methylated genes between high- and low-exposure groups. Through pathway analysis, 57 significant gene ontology enrichment terms were identified, including glutathione derivative biosynthetic process and nervous system development; thereby potentially bringing mechanistic insight into the toxic effects of lead exposure. The most recent study of lead exposure upon the epigenome is that of Devóz et al. (75), who examined promoter methylation of four genes related to glutathione activity: Glutamate-Cysteine Ligase Catalytic Subunit (*GCLC)*; Glutathione Peroxidase 1 (*GPX1)*; Glutathione-Disulfide Reductase (*GSR);* and *GSTP1*. In workers exposed to lead, high blood lead levels were associated with reduced *GCLC* and *GSTP1* methylation, potentially supporting the findings of Zhang et al. and indicating that this pathway may be crucial to understanding biological response to lead exposure. The two most recent research works about lead exposure and DNA methlyation, are the one from Issah et al. (102), where they studied global DNA methylation (using LINE-1 as an indicator) in electronic waste workers exposed to lead; they did not find statistically significant differences between exposed group (*n* = 100) vs. control group (*n* = 51). Finally, Meng et al. (76), in an effort of relating DNA damage induced by lead in exposed workers, analyzed methylation using the 850K Beadchip platform, comparing methylation in workers with an already established DNA-damage biomarker, with workers without such damage as well as controls; first, differentially methylated positions (DMPs) between the controls and the exposed workers were identified, In addition, DMPs were identified between the DNA-undamaged and DNA-damaged workers. Methylation levels of four candidate genes were measured by pyrosequencing in an independent sample set; the result of comparisons between the controls and the lead-exposed workers show that DMPs were significantly enriched in genes related to nerve conduction and cell cycle. Between DNA-damaged group and DNA-undamaged group, differentially methylated genes were enriched in the pathways related to cell cycle and DNA integrity checkpoints.

### Chromium VI

Chromium VI (Cr VI) is carcinogenic and targets the respiratory system, kidneys, liver, skin, and eyes. The first study of DNA methylation and chromium exposure is from 2012, when Wang et al. recruited 115 workers from chromate producing facilities and examined global DNA methylation patterns. They observed global hypomethylation in exposed workers, which they hypothesized to be the product of depleted serum folate levels that were also observed with exposure. In 2016, Yang et al. (77) performed the first study of mitochondrial DNA methylation in relation to chromium exposure, which revealed *MT-TF* and *MT-RNR1* hypomethylation and lower levels of methylation of these genes in workers with highest blood chromium levels. Most recently, in 2018 Hu et al. (78) examined promoter methylation of genes implicated in DNA damage response and repair. They observed *OGG1, MGMT*, and *RAD51* hypermethylation in exposed workers, with a positive correlation observed between exposure level and methylation, and a negative correlation between methylation and expression. These findings may suggest that chromium exposure leads to down-regulation of tumor-suppressor genes, thereby providing mechanistic insight into the carcinogenic processes associated with exposure.

### Welding fumes exposure

Prolonged exposure to welding fumes may cause lung damage and various types of cancer, including that of the lung, larynx and urinary tract. Health effects from certain fumes may include metal fume fever, stomach ulcers, kidney damage and nervous system damage. Three studies have been performed that have examined DNA methylation changes in workers exposed to welding fumes. In the first of these, from 2015, Li et al. (80) studied welders from Sweden and assessed methylation in a non-specified regions of tumor-suppressor genes such as Homeobox A9 (*HOXA9)*, Short Stature Homeobox 2 (*SHOX2), CDKN2A, MGMT*, and *APC*. They found *APC* higher methylation in welders. This was also associated with wood burning at home and has been shown elsewhere to be associated with tobacco smoke (103), suggesting that different products of combustion may have a similar effect upon this gene. That same year, Hossain et al. investigated methylation of four CpG sites mapping to exon 2 of the *F2RL3* gene in 101 welders and 127 controls. They found hypomethylation at one CpG site, while the number of years of welding work was associated with hypomethylation of another. As the *F2RL3* gene has been most widely studied in relation to tobacco smoke exposure, this study provides further evidence of overlap between the epigenetic changes associated with different types of combustion. Also in 2017, Xu et al. (81) investigated the impact upon the mitochondrial epigenome. They reported hypomethylation of the regulatory D-Loop region and of the *MT-TF* gene, with their methylation associated with particle-containing welding fumes exposure. Very recently, Dauter et al. (82) in a longitudinal study where 78 welders and 96 controls were examined on two occasions 6 years apart, measured DNA methylation of CpG sites within the cancer-related genes *AHRR, F2RL3*, and *B3GNTL1* by pyrosequencing. They report that, compared with controls, welders showed a significant decrease over time in DNA methylation of *B3GNTL1* CpG1 and CpG4. In addition, exposure to respirable dust and cumulative exposure was associated with a decrease in methylation of F2RL3 CpG2 among all welders.

### Coal

Many different disorders have been associated with exposure to coal, including pneumoconiosis, fibrosis, asbestosis, silicosis, emphysema, loss of lung function, and cancer. To date, there have been two studies of the effect of coal upon the human epigenome. The first, by de Souza et al. (84), examined global DNA methylation in coal miners, which revealed global hypermethylation in exposed workers compared with controls. In 2020, the same group of researchers investigated possible relationships between global methylation and polymorphisms in xenobiotic-metabolizing enzymes. Interestingly, they reported that variants within the *CYP1A1* gene were associated with lower global methylation levels in coal-exposed individuals, but no significant impact was observed for variants in glutathione-associated genes or others.

### Radiation

Radiation is a well-known cause of cancer. For most individuals, exposure predominantly comes from natural sources, but some occupations can result in significantly higher exposure levels. In 2015, Lee et al. (86), studied methylation levels at the repetitive elements LINE-1 and *Sat-2* in nuclear power plant workers. Global DNA methylation levels were lower in radiation-exposed workers than in controls, with LINE-1 methylation negatively correlated with total cumulative radiation dose and with the presence of chromosomal abnormalities. One year later, Kuzmina et al. (87) examined *RASSF1A, CDKN2A*, and *GSTP1* promoter methylation in individuals with a history of exposure to ionizing radiation (including those involved with clean-up of the Chernobyl nuclear plant) in comparison to controls. They observed that *GSTP1* and *p16* hypermethylation were correlated with exposure. Expanding on their work in relation to the long-term effects of radiation exposure, Kuzmina et al. (88) performed a validation study in a separate cohort of 49 workers from another nuclear plant. They again reported associations with methylation of the *GSTP1* and *p16* genes, and they additionally observed hypermethylation of the *TP53* and *SOD3* gene promoters in comparison to non-exposed individuals.

In 2019, Cho et al. (89), examined global methylation in 40 radiographers compared to 28 controls *via* both LINE-1 assay and luminometric methylation assay (LUMA). They observed global hypomethylation in the exposed group, with methylation of LINE-1 elements associated with cumulative radiation dosage and also associated with chromosomal abnormalities. In the same year, Chen et al. (90) studied 117 physicians exposed to low doses of ionizing radiation. Although marginal, they observed global hypomethylation, suggesting an impact of even comparatively low dosages upon the epigenome.

### Nanoparticles

The impact of nanoparticles upon the epigenome is a field that is only now beginning to develop. The first publication on this topic, published in 2020 by Rossnerova et al. (92), used the Illumina EPIC (850 K) microarray to profile DNA methylation in a group of 20 long-term exposed research workers and 20 controls. They reported hypomethylation at 341 CpG sites and hypermethylation of 364, with leading hits mapping to genes involved in pathways such as immune function and xenobiotic detoxification. More recently, in 2021 Rossnerova et al. (91) reported their findings from a longitudinal study where they analyzed DNA methylation in leukocytes from 10 participants occupationally exposed to nanoparticles and 4 non-exposed controls over the course of 4 years. While they observed methylation changes within both groups, these were more pronounced in the exposed subjects, which they hypothesized to be part of adaptation to chronic nanoparticle exposure.

### Formaldehyde

To date, there have been only two studies of the effects of formaldehyde upon the epigenome. In 2019 Barbosa et al. (104) analyzed global methylation in 49 beauty salon workers, which revealed hypermethylation in exposed workers. More recently, van der Laan et al. (20) measured epigenetic age acceleration in 31 Chinese workers occupationally exposed to formaldehyde in comparison to 39 non-exposed controls, but they did not observe statistically significant differences.

### Trichloroethylene

Phillips et al. (93) performed epigenome-wide analysis of DNA methylation using the 450 k microarray in 67 workers exposed to TCE (30 low exposure and 37 high exposure) compared to 73 non-exposed control subjects. Interestingly, they reported increased variation in methylation patterns among exposed individuals, and perhaps on account of this only 25 CpG sites were determined to show significant differences in comparison to controls. Notably, the Tripartite Motif Containing 68 (*TRIM68)* gene promoter was hypomethylated with TCE exposure, although the implications of this finding are not currently clear. The aforementioned study by van der Laan et al. (20) of epigenetic age in response to environmental exposures also examined the impact of TCE, and in contrast to formaldehyde, it was demonstrated that exposure to TCE increased age acceleration. Together, these studies may suggest an impact of TCE upon the epigenome, but that this appears to be broad and variable, as opposed to inducing gene-specific changes.

### Firefighters

Chronic exposure to smoke is one of the main risks for firefighters, and one study of the impact of their exposure to PAHs was described in a previous section. Here, we include two studies that focus on their profession, rather than exposure to a specific compound(s). The first of these, from Zhou et al. (94), examined differences between newly recruited firefighters and incumbents, using the Illumina Infinium EPIC microarray. They identified four CpGs, including three mapping to the Yip1 Domain Family Member 6 (*YIPF6)*, Mercaptopyruvate Sulfurtransferase (*MPST)* and PC-esterase domain-containing protein 1B (*PCED1B)* genes, with significant changes in methylation, with three showing higher methylation in incumbents. Global methylation assessment was also able to discriminate between incumbents (including by years of service) and new recruits. The other study performed in firefighters is the very recent publication by Goodrich et al. (95). In this work, once again the Illumina EPIC microarray was used, this time to compare 31 Hispanic white and 163 non-Hispanic white firefighters. Five xenobiotic metabolizing genes were reported to be differentially methylated, including three CpG sites within the promoter region of the biotransformation gene Sulfotransferase C2 (*SULT1C2)* having lower methylation in Hispanic compared to non-Hispanic firefighters. In 2022, the same group of scientists, performed a repeated measures methylation study in firefighters, using a platform that interrogates up to 850,000 methylation sites, measuring methylation levels prior to live fire training and 20–37 months later. They report 680 CpG sites with altered methylation, including 60 with at least a 5% methylation difference at follow-up; genes with differentially methylated CpG sites were enriched in biological pathways related to cancers, neurological function, cell signaling and transcription regulation.

### Hairdressers

Working as a hairdresser has been associated with increased risk for cancer, particularly bladder cancer. We found two publications performed in these workers that analyzed DNA methylation. Li et al. (97) recruited 295 hairdressers and analyzed DNA methylation of the following genes: *APC*; *CDKN2A*; Death Associated Protein Kinase 1 (*DAPK1)*; *GSTP1*; *MGMT*; POU domain, class 4, transcription factor 2 (*POU4F2)*; *RASSF1A*; RUNX Family Transcription Factor 3 (*RUNX3);* and Twist Family BHLH Transcription Factor 1 (*TWIST1)*. They did not observe statistically significant differences between groups, but they reported that “hair waving” was associated with less frequent *CDKN2A* methylation. In 2019, Liljedahl et al. (98) performed gene-specific analysis of DNA methylation in the same cohort of workers studied in the former work. This time they analyzed a greater number of genes: *APC*; *CDKN2A*; *DAPK1*; Docking Protein 1 (*DOK1)*; *GSTP1*; *MGMT*; *POU4F2*; *RASSF1A*; *RUNX3*; *SHOX2;* and *TWIST1*. They confirmed no significant differences between groups, but now described that carriers of genetic variants within the *FLG* gene showed higher methylation of *CDKN2A*.

### Vinyl chloride

Wu et al. (99) studied methylation of the *MGMT* and *hMLH1* gene promoters in 101 workers exposed to this compound. *MGMT* was demonstrated to be hypomethylated, which was only observed in individuals showing chromosomal abnormalities. In 2022, Zhao et al. (100) performing a whole-genome bisulfite sequencing in peripheral leukocytes from three workers exposed to vynil chloride and their respective controls, found that exposed workers showed A total of 9,534 differentially methylated regions (DMRs), of which 4,816 were hypomethylated and 4,718 were hypermethylated. After that first approach, they also selected 50 participants from each group and then methylation of differentially methylated genes (DMGs) found were verified by methylation-specific PCR (MSP) and agarose gel electrophoresis: the coincidence rate was 60–100%. The most involved pathways from the differentially methylated regions observed, corresponded to cancer, neuroactive ligand-receptor interaction, and axon guidance.

### Miscellaneous

In this section, we review all those research works for chemical compounds that comprise the sole study of each.

#### 1,6-hexamethylene diisocyanate

In 2014, Nylander-French et al. (105) studied 20 automotive spray-painters and performed epigenome-wide analysis by 450K microarray. They reported that methylation at 114 CpG loci were significantly associated with the exposure and urine biomarker levels, with Latrophilin 3 (*LPHN3)* and Scavenger Receptor Class A Member 5 (*SCARA5)* appearing to be of particular interest.

#### Aluminum

Yang et al. (106) measured DNA global methylation in aluminum-exposed pot room workers, without inclusion of a control group. They observed significant decreases in global DNA methylation with increasing levels of exposure.

#### Arsenic

Janasik et al. (107) recruited 61 occupationally-exposed men and assessed global methylation as well as promoter methylation of the Nuclear Factor Erythroid 2–related Factor 2 (*NRF2)* and Kelch-like ECH-associated protein 1 (*KEAP1)* genes. They observed both global and gene-specific hypermethylation in the exposed group, with levels of arsenic exposure positively correlated with global DNA methylation.

#### Asbestos

Yu et al. (108) analyzed 47 healthy subjects with exposure to asbestos and compared them to both a control group and to 52 individuals diagnosed with benign asbestos-related disorders. They observed that the groups with asbestos exposure and with benign disorders both showed decreased global DNA methylation levels compared with controls, and with no significant difference between asbestos-exposed individuals with and without benign disorders.

#### Carbon nanotubes

Ghosh et al. (109) studied multi-wall carbon nanotubes exposed workers, analyzing both global methylation and promoter methylation for the following genes: DNA methyltransferase 1 (*DNMT1)*; Histone Deacetylase 4 (*HDAC4)*; Nuclear Protein, Coactivator Of Histone Transcription (*NPAT/ATM);* and Shikimate Kinase 1 (*SKI)*. They did not observe an impact upon global methylation, but the *DNMT1* promoter was hypermethylated while *HDAC* and *NPATM* were hypermethylated too. Interestingly, *SKI* showed both gains and losses of methylation at different CpG sites.

#### Coal tar

Wang et al. (110) recruited 180 workers exposed to coal tar pitch and examined methylation of the *p16*, Fragile Histidine Triad Diadenosine Triphosphatase (*FHIT)* and *RASSF1A* gene promoters. They did not find significant differences between groups, although they observed that *p16* methylation was higher in exposed individuals when analysis was restricted to younger individuals (<40).

#### Construction environment

Silva et al. (111) studied *CDKN2A, MLH1*, and *APC* promoter methylation and LINE-1 as a surrogate marker of global methylation in 59 workers exposed to the construction environment and in 49 unexposed workers. They observed higher average levels of methylation for all three genes and hypomethylation of the LINE-1.

#### Diesel exhaust

Shen et al. (112) analyzed promoter methylation of *p16, RASSF1A*, and *MGMT* in workers with occupational exposure to diesel engine exhaust. All three were reported to be hypomethylated and showed with negative correlations with the presence of DNA adducts.

#### Diisocyanate

Ouyang et al. (113), studied promoter methylation in 131 workers, with hypermethylation of *GSTM1, DUSP22, IFN-*γ, and *IL-4*. *IFN-*γ observed in the exposed group.

#### Mineral dust

In 2019, van der Plaat et al. (114) analyzed, by means of the Illumina 450K microarray platform, differentially methylated regions in exposed workers. Exposure to gasses/fumes and to mineral dust were associated with 14 and 7 differentially methylated regions (DMRs), respectively. Three of these showed changes in responses to both dust and to gasses and fumes, which mapped to the 60S Ribosomal Protein L1 (*RPL1, one* DMR) and Long Intergenic Non-Protein Coding RNA 2169 (*LINC02169*, two DMRs) genes. Many of the observed changes were demonstrated to impact upon gene expression, thereby potentially providing mechanistic insight into the impact of this exposure.

#### Manganese

In 2015, Searles Nielsen et al. (115) assessed DNA methylation at three CpG sites in first exon of the *NOS2* gene in blood taken from 201 welders, which revealed hypomethylation in exposed workers.

#### Nickel

Yang et al. (116) studied gene-specific methylation of the *p15* and *p16* genes in nickel exposed workers. The *p15* gene was observed to be hypermethylated, with workers having higher urinary nickel at increased risk of hypermethylation.

#### Polychlorinated biphenyl

Wang et al. (117), using an epigenome-wide approach, investigated methylation profiles in male employees in an e-waste dismantling area from China. Exposure was associated with 391 hypomethylation sites and 553 hypermethylation sites, with gene expression demonstrated to be affected for ten genes.

## Discussion

In this review, we have summarized current knowledge on the impact of toxic agents upon the epigenome (DNA methylation specifically) in the blood of occupationally-exposed workers. Occupational exposures are particularly different, compared with incidental exposure in the general population, on account of the frequency, duration and dosage of exposure, with individuals often continuously exposed for 8 h or more for 5 or 6 days at a constant level. These unique characteristics of occupational exposures make such individuals an almost controllable model of exposure, compared to incidental (and often non-continuous) exposures of varying degrees in general population. Subsequently, the study of occupational exposures, as in these collated 116 research papers, can provide strong evidence and great insight into epigenetic mechanisms of toxicity. As summarized in Table 1, most of the chemicals studied are related to carcinogenesis, with benzene exposure the most widely studied. When taking into consideration that our review has exclusively focused upon studies of apparently healthy individuals and not those who have already developed disease, all statistically significant results in exposed groups may be considered as early modifications related to disease onset. While epigenome-wide analysis is increasingly common, the most widely-used platform to date has been bisulfite pyrosequencing, which has typically been used to assess global DNA methylation via LINE-1 or *Alu* assay, but also to focus upon genes related with DNA repair and other tumor-suppressor genes. We regret that few studies have examined the mitochondrial epigenome, and this is clearly an area that warrants further study. Almost 90% of the papers reported significant differences in an exposed group compared with controls, with global hypomethylation observed almost invariably with a wide range of exposures, and promoter hypermethylation also being commonly reported. Only three papers reported concurrent hypomethylation and hypermethylation of different CpG sites within the same gene or locus, of which two reported hypermethylation in the promoter region and hypomethylation within the 5′UTR of the same gene. As methylation within the 5′UTR is very frequently associated with gene transcription, such observations require further work to understand the potential functional consequences. At this point, results from epigenome-wide studies cannot be readily compared due to the complexity of analytical approaches and the comparatively small number of studies. An important question within the field is the persistence of the observed changes (or lack thereof), and therefore it is worth to highlight and to contrast the results from the only two longitudinal studies where authors performed repeated methylation measures. Ren et al. (14) monitored 35 workers exposed to benzene, comparing their methylation levels in 2009 and later in 2013. During this time their exposure levels decreased, and subsequently they reported that LINE-1 methylation levels increased in 2013 compared to 2009 while *hMLH1* methylation levels decreased. On the other hand, Rossnerova et al. (91) reported what they call a “higher stability” in five differentially methylated CpG sites evaluated four times annually in 10 workers exposed to nanoparticles, suggesting an enforcement of epigenetic changes through continuous exposure. As these results suggest dynamic changes in DNA methylation in response to exposure levels, which are therefore likely to fluctuate over the lifecourse, they raise some important questions: how transitory are the observed global hypomethylation and gene-specific hypermethylation within exposed individuals? If they are more dynamic, what would measurement of DNA methylation at a single timepoint tell us about the real risk to develop disease? It should be noted that the observations from the two aforementioned studies were in genes from very different cellular pathways, in addition to the toxicant of exposure being different. Another important contribution are those results showing correlation of methylation levels with an already established clinical or diagnostic endpoint, such as neutrophil abundance in the blood or cell/tissue damage endpoints such as cytokinesis-block micronucleus.

At light of all considerations mentioned in the former paragraph, regarding the few longitudinal studies, and also that the epigenetic modifications observed in a certain window of time may be transitory, we think that, with the results obtained in the 116 papers reviewed, we cannot give an insight about the possible consequences of such DNA alterations, since this would be speculative. Based also in the former commentary, we think that results obtained in the several studies do not allow as to claim that observed changes in DNA methylation may be considered as functional or pathological correlates. We consider that, the interest for investigating global and/or promoter hypermethylation as a result of toxicant exposures, mainly for those toxicants considered carcinogenic in humans, comes from the observations in tumors and/or already cancerous cells, where these two modifications were published first in the early eighties and confirmed in the nineties by several studies. However, two considerations are important to interpret these results obtained in occupationally-exposed workers: (1) they are healthy people, and (2) DNA methylation was performed in peripheral blood cells; then, we cannot generalize in the sense that all epigenetic modifications found in blood cells reflects the changes related with carcinogenesis that may occur in other target cells. For instance, in this review we have found that, occupational exposure to arsenic (a known carcinogenic agent) caused global hypermethylation in blood cells, while trichloroethylene and diesel exhaust exposure (also related with cancer development in different tissues) caused gene promoter hypomethylation; on the contrary, 95% of the studies in workers exposed to benzene report hypomethylation of Alu and LINE-1, as surrogate markers of global methylation; however, benzene, contrary to arsenic, trichloroethylene and diesel exhaust, does affect cells from the hematopoietic system. This tells us about the lack of homogeneity for the different results, and therefore, so far, we cannot say that modifications observed in DNA methylation are predictive of certain disease development.

## Conclusion and recommendations

Over the course of 15 years, many and varied results have been published in the toxicomethylomics field, specifically in blood cells from occupational-exposed individuals. It is difficult to generalize the observations those studies have described, mostly because of the cross-sectional nature of the existing publications and their heterogeneous nature. We are still far from considering methylation changes (either global or gene-specific) as a biomarker of effect for most chemical exposures. Even though global hypomethylation and gene-specific promoter hypermethylation (albeit for different genes) may be considered as consistent observations for different exposures, to date there has been no establishment of functional or pathological correlates for those epigenetic modifications. We suggest to create a global network of experts for co-ordinated examination of genes and pathways for the same chemical exposure, with particular focus on longitudinal studies, in order to help bring consensus for how epigenetic changes may be implicated in the mechanisms of toxicity for disease development. Furthermore, we also propose that more studies should examine mitochondrial DNA, since this organelle is very susceptible to early damage, mostly from reactive oxygen species, which is a common toxicological alteration related with all mechanisms of toxicity.

## Author contributions

OJ-G: concept design, paper writing, and bibliographic research. LG: supervision and writing. MG: writing and concept design. TB: style correction and concept design. All authors contributed to the article and approved the submitted version.
